# Supplementary material for: Identification of Freezing-Responsive microRNAs and Their Targets in Chinese Jujube by Small RNA and Degradome Sequencing
Source: Int J Mol Sci. 2026 Sep 4;27(17):7912. doi: 10.3390/ijms27177912 (PMC13566890; doi:10.3390/ijms27177912)
Supplement: Supplementary file 1 [file ijms-27-07912-s001.zip › Supplementary Material S2/novel_miR_16.pdf]

Provisional ID	:	novel_miR_16
Score total	:	338.4
Score for star read(s)	:	3.9
Score for read counts	:	336.6
Score for mfe	:	0.1
Score for randfold	:	-2.2
Score for cons. seed	:	
Total read count	:	672
Mature read count	:	473
Loop read count	:	108
Star read count	:	91

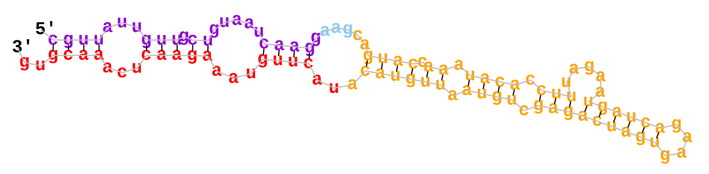

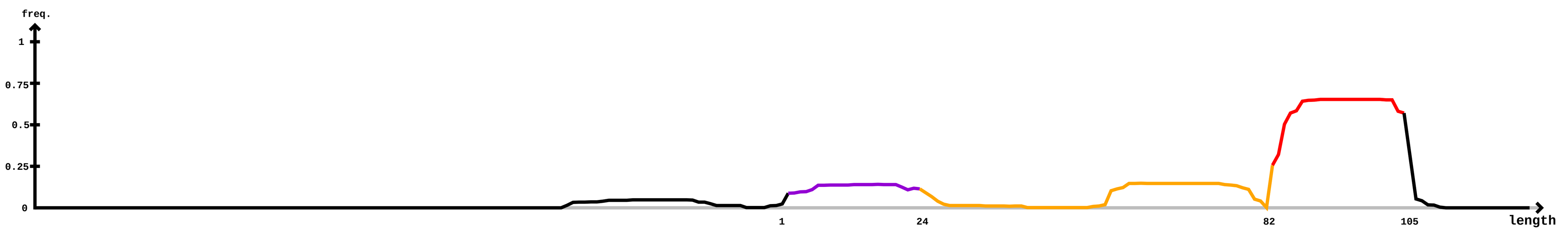

Star Mature

```
5'- aaaacaaauauaaaauugauucaaccccaagccauuugaauguaguggauaacaguggagcccgagaauuaaugaguauuugaaucguaggagcuaguaaucgacgauaugcucauauuggugacguuauuguugcuguaaucaaggaagcaguaccaaauacaccuuuagaaugaucagaagugaucagagcuguaauuguacauacuuguaaagaacucaaacgugagaacgauaugaucauacaaca  -3'  obs
    aaaacaaauauaaaauugauucaaccccaagccauuugaauguaguggauaacaguggagcccgagaauuaaugaguauuugaaucguaggagcuaguaaucgacgauaugcucauauuggugacguuauuguugcuguaaucaaggaagcaguaccaaauacaccuuuagaaugaucagaagugaucagagcuguaauuguacauacuuguaaagaacucaaacgugagaacgauaugaucauacaaca       exp
    ....((((((...(((((((...((((..(((((.......)))))......))).).....)))))))))..)))))))..((((..((........)).))))((((.((((((((...(((((...(((.((.....((((......((((.((.((((.(((.....((((((....)))))))))).)))).))))))...))))...)))))...)))))).....)))))))).)))).....  reads  mm  sample
    .......................................................................................uaggagcuaguaaucgacgaua.............................................................................................................................................  2  0  LT-1
    .......................................................................................uaggagcuaguaaucgacgaua.............................................................................................................................................  2  0  JCK-2
    .......................................................................................uaggagcuaguaaucgacgauaug...........................................................................................................................................  1  0  JCK-2
    .......................................................................................uaggagcuaguaaucgacgauaugc..........................................................................................................................................  6  0  LCK-3
    ........................................................................................aggagcuaguaaucgacgau..............................................................................................................................................  1  0  LT-3
    ........................................................................................aggagcuaguaaucgacgaua.............................................................................................................................................  3  0  LT-2
    ........................................................................................aggagcuaguaaucgacgaua.............................................................................................................................................  1  0  JCK-1
    ........................................................................................aggagcuaguaaucgacgaua.............................................................................................................................................  1  0  JCK-3
    ........................................................................................aggagcuaguaaucgacgauauU...........................................................................................................................................  1  1  LT-2
    ........................................................................................aggagcuaguaaucAacgauaug...........................................................................................................................................  1  1  JCK-2
    ........................................................................................aggagcuaguaaucgacgauaug...........................................................................................................................................  1  0  JCK-3
    ........................................................................................aggagcuaguaaucgacgauaug...........................................................................................................................................  1  0  LT-1
    ........................................................................................aggagcuaguaaucAacgauaug...........................................................................................................................................  1  1  JCK-3
    ........................................................................................aggagcuaguaaucgacgauaugc..........................................................................................................................................  2  0  LT-2
    .........................................................................................ggagcuaguaaucgacgauaugc..........................................................................................................................................  1  0  JCK-2
    ...........................................................................................agcuaguaaucgacgauaug...........................................................................................................................................  1  0  LT-3
    .............................................................................................cuaguaaucgacgauaugcucaua.....................................................................................................................................  2  0  JCK-3
    .............................................................................................cuaguaaucgacgauaugcucaua.....................................................................................................................................  1  0  JCK-2
    ..............................................................................................uaguaaucgacgauaugcucaua.....................................................................................................................................  1  0  LT-1
    ..............................................................................................uaguaaucgacgauaugcucaua.....................................................................................................................................  3  0  LT-2
    ..................................................................................................aaucgacgauaugcucaua.....................................................................................................................................  1  0  JCK-2
    ..................................................................................................aaucgacgauaugcucaua.....................................................................................................................................  1  0  JCK-3
    ................................................................................................................ucauauuggugacguuauuguugcugua..............................................................................................................  1  0  LT-1
    .........................................................................................................................ugacguuauuguugcuguaauca..........................................................................................................  2  0  JCK-2
    .........................................................................................................................ugacguuauuguugcuguaauca..........................................................................................................  3  0  JCK-3
    .........................................................................................................................ugacguuauuguugcuguaauca..........................................................................................................  3  0  JCK-1
    ..........................................................................................................................gacguuauuguugcuguaaucaaggaC.....................................................................................................  1  1  LT-1
    ...........................................................................................................................acguuauuguugcuguaauca..........................................................................................................  1  0  LT-3
    ...........................................................................................................................acguuauuguugcuguaaucaag........................................................................................................  2  0  LT-3
    ...........................................................................................................................acguuauuguugcuguaaucaagg.......................................................................................................  1  0  JCK-2
    ...........................................................................................................................acguuauuguugcuguaaucaagg.......................................................................................................  1  0  JCK-1
    ...........................................................................................................................acguuauuguugcuguaaucaaggaag....................................................................................................  2  0  LCK-3
    ............................................................................................................................cguuauuguugcuguaauU...........................................................................................................  10  1  LCK-3
```

Star **Mature**

aaaacaaauauaaaauugauucaaccccaagccauuugaauguaguggauaacaguggagcccgagaauuaaugaguauuugaaucguaggagcuaguaaucgacgauaugcucauauuggugacguuauuguugcuguaaucaaggaagcaguaccaaauacaccuuuagaaugaucagaagugaucagagcuguaauuguacauacuuguaaagaacucaaacgugagaacgauaugaucauacaaca

cguuauuguugcuguaauc	1	0	LT-3
cguuauuguugcuguaaucaag	1	0	JCK-3
cguuauuguugcuguaaucaagg	4	0	JCK-1
cguuCuuguugcuguaaucaagg	1	1	LCK-3
cguuauuguugcuguaaucaagg	15	0	LCK-3
cguuauuguugcuguaaucaagg	2	0	JCK-2
cguuauuguugcuguaaucaagga	4	0	LCK-3
cguuauuguugcuguaaucaagga	1	0	JCK-2
cguuauuguugcuguaaucaaggaag	2	0	LT-1
cguuauuguugcuguaaucaaggaagc	1	0	LCK-3
cguuauuguugcuguaaucaaggaagc	1	0	JT-2
cguuauuguugcuguaaucaaggaagc	2	0	JT-3
cguuauuguugcuguaaucaaggaagc	1	0	LT-2
cguuauuguugcuguaaucaaggaagc	1	0	LT-1
guuauuguugcuguaaucaagga	1	0	LT-1
uuauuguugcuguaauca	3	0	LCK-3
uuauuguugcuguaaucaagg	1	0	LT-3
uuauuguugcuguaaucaaggaag	1	0	LT-1
uauuguugcuguaaucaagg	1	0	LT-3
auuguugcuguaaucaagga	2	0	LT-3
auuguugcuguaaucaaggaa	2	0	JCK-1
auuguugcuguaaucaaggaag	5	0	LCK-3
uuguugcuguaaucaaggaa	7	0	JT-2
uuguugcuguaaucaaggaa	9	0	JT-3
uuguugcuguaaucaaggaag	1	0	LT-3
uuguugcuguaaucaaggaag	2	0	LT-2
guugcuguaaucaaggaa	1	0	LT-1
cuguaaucaaggaagcaguacc	1	0	JCK-3
cuguaaucaaggaagcaguacc	1	0	JCK-1
aaucaaggaagcaguaccaaau	1	0	LT-2
gAaagcaguaccaaauaca	7	1	LCK-3
caccuAuagaaugaucagaagu	1	1	LT-1
aucagaagugaucagagcuguaau	2	0	JCK-2
aucagaagugaucagagcuguaau	1	0	JCK-3
aucagaagugaucagagcuguaauuguac	1	0	JCK-1
aucagaagugaucagagcuguaauuguac	1	0	JCK-2
ucagaagugaucagagcugua	1	0	LT-1
ucagaagugaucagagcugua	1	0	JCK-3
cagaagugaucagagcugua	2	0	LT-2
cagaagugaucagagcuguaauu	1	0	JCK-2
cagaagugaucagagcuguaauu	1	0	JCK-1
cagaagugaucagagcuguaauu	1	0	JCK-3
cagaagugaucagagcuguaauuC	1	1	JCK-3
agaagugaucagagcuguaauu	2	0	JCK-2
agaagugaucagagcuguaauu	2	0	JCK-3
agaagugaucagagcuguaauu	1	0	LT-1
agaagugaucagagcuguaauug	3	0	JCK-2
agaagugaucagagcuguaauug	1	0	JCK-1
agaagugaucagagcuguaauug	1	0	JCK-3
agaagugaucagagcuguaauugu	9	0	JCK-2
agaagugaucagagcuguaauugu	8	0	JT-3
agaagugaucagagcuguaauugu	10	0	JCK-3
agaagugaucagagcuguaauugu	4	0	JT-2
agaagugaucagagcuguaauugu	8	0	JCK-1
aNaagugaucagagcuguaauugu	1	1	JCK-2
agaagugaucagagcuguaauuguac	4	0	JT-2
agaagugaucagagcuguaauuguac	1	0	JCK-3
agCagugaucagagcuguaauuguac	1	1	JT-3
agaagugaucagagcuguaauuguac	4	0	JT-3
agaagugaucagagcuguaauuguac	1	0	JCK-2
gaagugaucagagcugua	1	0	LT-2
gaagugaucagagcuguaa	2	0	JCK-1
gaagugaucagagcuguaauuguac	2	0	JCK-1
gaagugaucagagcuguaauuguac	1	0	LT-2
gaagugaucagagcuguaauuguac	2	0	JCK-2
aagugaucagaAcuguaauug	1	1	LT-1
aagugaucagagcuguaauugu	2	0	JCK-2
aagugaucagagcuguaauuguG	1	1	JCK-1
aagugaucagagcuguaauugua	1	0	JCK-2
aagugaucagagcuguaauuguG	1	1	JCK-2

```
                                                                                                                            Star                                                                             Mature
aaaacaaauauaaaauugauucaaccccaagccauuugaauguaguggauaacaguggagcccgagaauuaaugaguauuugaaucguaggagcuaguaaucgacgauaugcucauauuggugacguuauuguugcuguaaucaaggaagcaguaccaaauacaccuuuagaaugaucagaagugaucagagcuguaauuguacauacuuguaaagaacucaaacgugagaacgauaugaucauacaaca
.....................................................................................................................................................................................agugaucagagcuguaauu..................................................   1   0   LT-1
.....................................................................................................................................................................................agugaucagagcuguaauugu................................................   1   0   LT-3
.....................................................................................................................................................................................agugaucagagcuguaauugua...............................................   2   0   JT-3
.....................................................................................................................................................................................agugaucagagcuguaauuguU...............................................   1   1   JCK-1
.....................................................................................................................................................................................agugaucagagcuguaauuguU...............................................   2   1   JCK-3
.....................................................................................................................................................................................agugaucagagcuguaauuguac..............................................   2   0   LT-1
.....................................................................................................................................................................................agugaucagagcuguaauuguac..............................................   6   0   JCK-2
.....................................................................................................................................................................................agugaucagagcuguaauuguac..............................................   3   0   JCK-3
.......................................................................................................................................................................................ugaucagagcuguaauugua...............................................   1   0   LT-1
...........................................................................................................................................................................................................cauacuuAuaaagaacucaaacg........................   1   1   LT-3
.............................................................................................................................................................................................................uacuuguaaagaacucaaa..........................   2   0   LT-3
.............................................................................................................................................................................................................uacuuguaaagaacucaaacg........................   24  0   LCK-2
.............................................................................................................................................................................................................uacuuguaaagaacucaaacg........................   23  0   LCK-1
.............................................................................................................................................................................................................uacuuguaaagaacucaaacgu.......................   1   0   LT-3
.............................................................................................................................................................................................................uacuuguaaagaacucaaacgu.......................   2   0   LT-1
.............................................................................................................................................................................................................uacuuguaaagaacucaaacgug......................   18  0   LT-2
.............................................................................................................................................................................................................uacuuguaaagaacucaaacgug......................   6   0   LT-1
.............................................................................................................................................................................................................uCcuuguaaagaacucaaacgug......................   1   1   LCK-3
.............................................................................................................................................................................................................uacuuguaaagaacucaaacgug......................   11  0   LT-3
.............................................................................................................................................................................................................uacuuguaaaUaacucaaacgug......................   1   1   LT-3
.............................................................................................................................................................................................................uacuuguaaagaacucaGacgug......................   2   1   JCK-1
.............................................................................................................................................................................................................uacuuguaaagaacucaaacgug......................   6   0   JT-3
.............................................................................................................................................................................................................uacuuguaaagaacucaaacgug......................   9   0   JCK-1
.............................................................................................................................................................................................................uacuuguaaagaacucaaacgug......................   40  0   LCK-3
.............................................................................................................................................................................................................uacuuguaaagaacucaaacgug......................   11  0   JCK-2
.............................................................................................................................................................................................................uacuuguaaagaacucaaacgug......................   8   0   JT-2
.............................................................................................................................................................................................................uacuuguaaagaacucaaacgug......................   4   0   JCK-3
.............................................................................................................................................................................................................uacuuguaaagaacucaaacguga.....................   1   0   JCK-3
.............................................................................................................................................................................................................uacuuguaaagaacucaaacguga.....................   4   0   JT-2
.............................................................................................................................................................................................................uacuuguaaagaacucaaacguga.....................   4   0   JT-3
.............................................................................................................................................................................................................uacuuguaaagaacucaaacguga.....................   2   0   JCK-2
.............................................................................................................................................................................................................uacuuguaaagaacucaaacgugag....................   3   0   JCK-2
.............................................................................................................................................................................................................uacuuguaaagaacucaaacgugag....................   2   0   JCK-3
.............................................................................................................................................................................................................uacuuguaaagaacucaaacgugag....................   1   0   JCK-1
..............................................................................................................................................................................................................acuuguaaagaacucaaacgu.......................   2   0   JCK-3
..............................................................................................................................................................................................................acuuguaaagaacucaaacgu.......................   1   0   JCK-2
..............................................................................................................................................................................................................acuuguaaagaacucaaacgug......................   4   0   JCK-1
..............................................................................................................................................................................................................acuuguaaagaacucaaacgug......................   6   0   JCK-3
..............................................................................................................................................................................................................acuuguaaagaacucaaacgug......................   11  0   LT-2
..............................................................................................................................................................................................................acuuguaaagaacucaaacgug......................   1   0   JT-3
..............................................................................................................................................................................................................acuuguaaagaacucaaacgug......................   2   0   LT-1
..............................................................................................................................................................................................................acuuguaaagaacucaaacgug......................   7   0   LCK-3
..............................................................................................................................................................................................................acuuguaaagaacucaaacgug......................   2   0   LT-3
..............................................................................................................................................................................................................acuuguaaagaacucaaacgug......................   6   0   JCK-2
..............................................................................................................................................................................................................acuuguaaagaacucaaacgugag....................   2   0   LT-2
..............................................................................................................................................................................................................acuuguaaagaacucaaacgugaga...................   3   0   LT-2
...............................................................................................................................................................................................................cuuguaaagaacucaaacg........................   1   0   LT-3
...............................................................................................................................................................................................................cuuguaaagaacucaaacg........................   1   0   LT-1
...............................................................................................................................................................................................................cuuguaaagaacucaaacgu.......................   1   0   JCK-3
...............................................................................................................................................................................................................cuuguaaagaacucaaacgu.......................   1   0   JCK-1
...............................................................................................................................................................................................................cuuguaaagaacucaaacgug......................   5   0   JT-2
...............................................................................................................................................................................................................cuuguaaagaacucaaacgug......................   8   0   JCK-3
...............................................................................................................................................................................................................cuuguaaagaCcucaaacgug......................   1   1   LCK-3
...............................................................................................................................................................................................................cuuguaaagaacNcaaacgug......................   1   1   LCK-1
...............................................................................................................................................................................................................cuuguaaagaacucaaacgug......................   5   0   JT-3
...............................................................................................................................................................................................................cuuguaaagaacucaaacgug......................   4   0   LCK-3
...............................................................................................................................................................................................................cuuguaaagaacucaaacgug......................   21  0   LCK-2
...............................................................................................................................................................................................................cuuguaaagaacucaaacgug......................   10  0   JCK-2
...............................................................................................................................................................................................................cuuguaaagaCcucaaacgug......................   1   1   LT-1
...............................................................................................................................................................................................................cuuguaaagaacucaaacgug......................   7   0   LT-2
...............................................................................................................................................................................................................cuuguaaagaacucaaacgug......................   6   0   LT-3
...............................................................................................................................................................................................................cuuguaaagaacucaaacgug......................   4   0   JCK-1
...............................................................................................................................................................................................................cuuguaaagaacucaaacgug......................   38  0   LCK-1
...............................................................................................................................................................................................................cuuguaaagaacucaaacguga.....................   2   0   JCK-3
...............................................................................................................................................................................................................cuuguaaagaacucaaacgugU.....................   2   1   JCK-2
...............................................................................................................................................................................................................cuuguaaagaacucaaacguga.....................   2   0   JCK-1
...............................................................................................................................................................................................................cuuguaaagaacucaaacguga.....................   1   0   JCK-2
...............................................................................................................................................................................................................cuuguaaagaacucaaacgugU.....................   1   1   JCK-1
...............................................................................................................................................................................................................cuuguaaagaacucaaacgugU.....................   1   1   JCK-3
...............................................................................................................................................................................................................cuuguaaagaacucaaacgugaga...................   5   0   LCK-3
```

```
                                                                                                                              Star                                                                                 Mature
aaaacaaauauaaaauugauucaaccccaagccauuugaauguaguggauaacaguggagcccgagaauuaaugaguauuugaaucguaggagcuaguaaucgacgauaugcucauauuggugacguuauuguugcuguaaucaaggaagcaguaccaaauacaccuuuagaaugaucagaagugaucagagcuguaauuguacauacuuguaaagaacucaaacgugagaacgauaugaucauacaaca
...............................................................................................................................................................................................................cuuguaaagaacucaaacgugagaac.................   2   0   LCK-3
...............................................................................................................................................................................................................cuuguaaagaacucaaacgugagaac.................   1   0   JCK-2
...............................................................................................................................................................................................................cuuguaaagaacucaaacgugagaac.................   1   0   JCK-1
................................................................................................................................................................................................................uuguaaagaacucaaacgug......................   2   0   JCK-3
................................................................................................................................................................................................................uuguaaagaacucaaacgug......................   11  0   JT-1
................................................................................................................................................................................................................uuguaaagaacucaaacgug......................   2   0   LT-1
................................................................................................................................................................................................................uuguaaagaacucaaacgug......................   4   0   LCK-3
................................................................................................................................................................................................................uuguaaagaacucaaacgug......................   11  0   LT-2
................................................................................................................................................................................................................uuguaaagaacucaaacgug......................   3   0   LT-3
................................................................................................................................................................................................................uuguaaagaacucaaacgugU.....................   4   1   LT-2
................................................................................................................................................................................................................uuguaaagaacucaaacguga.....................   1   0   JCK-2
................................................................................................................................................................................................................uuguaaagaacucaaacguga.....................   2   0   JCK-1
................................................................................................................................................................................................................uuguaaagaacucaaacgugU.....................   2   1   JCK-1
................................................................................................................................................................................................................uuguaaagaacucaaacgugaga...................   3   0   LT-2
................................................................................................................................................................................................................uuguaaagaacucaaacgugagaac.................   4   0   LCK-3
.................................................................................................................................................................................................................uguaaagaacucaaacgug......................   1   0   JCK-2
.................................................................................................................................................................................................................uguaaagaacucaaacgug......................   9   0   JT-1
..................................................................................................................................................................................................................guaaagaacucaaacgug......................   4   0   LCK-3
..................................................................................................................................................................................................................guaaagaacucaaacgug......................   14  0   JT-1
..................................................................................................................................................................................................................guaaagaacucaaacgug......................   3   0   LT-1
..................................................................................................................................................................................................................guaaagaacucaaacgug......................   14  0   JCK-2
..................................................................................................................................................................................................................Auaaagaacucaaacgug......................   1   1   JCK-2
..................................................................................................................................................................................................................guaaagaacucaaacgugU.....................   2   1   LT-2
..................................................................................................................................................................................................................guaaagaacucaaacgugaga...................   1   0   LT-3
..................................................................................................................................................................................................................guaaagaacucaaacgugagaacg................   2   0   JCK-2
..................................................................................................................................................................................................................guaaagaacucaaacgugagaacg................   1   0   JCK-1
...................................................................................................................................................................................................................uaaagaacucaaacgugaga...................   4   0   LCK-3
....................................................................................................................................................................................................................aaagaacucaaacgugagaac.................   1   0   LT-1
.....................................................................................................................................................................................................................aagaacucaaacgugaUa...................   2   1   LT-2
.....................................................................................................................................................................................................................aagaacucaaacgugagaa..................   1   0   LT-1
```
